# Impact of Ketamine on Analgosedative Consumption in Critically Ill Patients: A Systematic Review and Meta-Analysis

**DOI:** 10.1177/10600280211069617

**Published:** 2022-01-26

**Authors:** Katalina Chan, Lisa D. Burry, Christopher Tse, Hannah Wunsch, Charmaine De Castro, David R. Williamson

**Affiliations:** 1 Institute of Health Policy, Management and Evaluation, University of Toronto, Toronto, ON, Canada; 2 Novo Nordisk Canada Inc, Mississauga, ON, Canada; 3 Department of Pharmacy and Medicine, Sinai Health System, Toronto, ON, Canada; 4 Leslie Dan Faculty of Pharmacy, University of Toronto, Toronto, ON, Canada; 5 Department of Pharmacy, Princess Margaret Hospital, Toronto, ON, Canada; 6 Department of Critical Care Medicine, Sunnybrook Health Sciences Centre, Toronto, ON, Canada; 7 Interdepartmental Division of Critical Care Medicine, Department of Anesthesiology and Pain Medicine, University of Toronto, Toronto, ON, Canada; 8 Sidney Liswood Health Sciences Library, Sinai Health System, Toronto, ON, Canada; 9 Faculté de Pharmacie, Université de Montréal, Montréal, QC, Canada; 10 Pharmacy Department and Research Center, CIUSSS du nord-de-l’Île-de-Montréal, Sacré-Cœur Hospital, Montréal, QC, Canada

**Keywords:** analgosedation, benzodiazepine, critical care, ketamine, opioid, sedative

## Abstract

**Objective:**

The aim of this study was to synthesize evidence available on continuous infusion ketamine versus nonketamine regimens for analgosedation in critically ill patients.

**Data sources:**

A search of MEDLINE, EMBASE, CINAHL, CDSR, and ClinicalTrials.gov was performed from database establishment to November 2021 using the following search terms: *critical care, ICU, ketamine, sedation*, and *anesthesia*. All studies included the primary outcome of interest: daily opioid and/or sedative consumption.

**Study selection and data extraction:**

Relevant human studies were considered. Randomized controlled trials (RCT), quasi-experimental studies, and observational cohort studies were eligible. Two reviewers independently screened articles, extracted data, and appraised studies using the Cochrane RoB and ROBINS-I tools.

**Data synthesis:**

A total of 13 RCTs, 5 retrospective, and 1 prospective cohort study were included (2255 participants). The primary analysis of six RCTs demonstrated reduced opioid consumption with ketamine regimens (n = 494 participants, −13.19 µg kg^−1^ h^−1^ morphine equivalents, 95% CI −22.10 to −4.28, *P* = 0.004). No significant difference was observed in sedative consumption, duration of mechanical ventilation (MV), ICU or hospital length of stay (LOS), intracranial pressure, and mortality. Small sample size of studies may have limited ability to detect true differences between groups.

**Relevance to patient care and clinical practice:**

This meta-analysis examining ketamine use in critically ill patients is the first restricting analysis to RCTs and includes up-to-date publication of trials. Findings may guide clinicians in consideration and dosing of ketamine for multimodal analgosedation.

**Conclusion:**

Results suggest ketamine as an adjunct analgosedative has the potential to reduce opioid exposure in postoperative and MV patients in the ICU. More RCTs are required before recommending routine use of ketamine in select populations.

## Introduction

Traditional critical care analgesic and sedative drugs require careful selection and titration to balance their efficacy and adverse effect profile as their use can result in extended mechanical ventilation (MV), prolonged stay, and long-term morbidity.^1,2^ Choice of agent is influenced by a number of factors.^3^

Opioids are a mainstay for analgosedation in the ICU^3^ but are limited by tolerance, hyperalgesia, reduced blood pressure, and risk of withdrawal^4^ or persistent use.^5^ Judicious use is favorable amidst a global opioid crisis that affects approximately 36.3 million people.^6^ Benzodiazepines, a frequently administered sedative, poses risks of respiratory and cardiovascular depression, delirium, and unintended oversedation from drug accumulation.^1^ Nonbenzodiazepine sedatives (propofol, dexmedetomidine) may be preferrable alternatives due to evidence of improved short-term outcomes (ICU length of stay [LOS], duration of MV, and delirium). However, they too are limited by the risk of hypotension, hemodynamic instability, and in the case of dexmedetomidine, cost.^2,7,8^

Multimodal analgesia can be used to minimize opioid use and optimize analgosedation.^3^ Ketamine is an attractive adjunct with both sedative and analgesic properties, quick onset of action, and limited bioaccumulation and rapid recovery.^9^ However, ketamine may precipitate psychomimetic adverse effects (e.g., hallucinations and nightmares) and at high doses, can also impact cardiovascular function, increasing blood pressure, heart rate, and arrhythmias.^7,9^ Due to the dose-dependent adverse effects, ketamine may be used more often as an opioid and sedative sparing agent in conjunction with other medications rather than as a solo agent.

The evidence to support ketamine use in the ICU is growing. It is hypothesized that ketamine administration in the critically ill may reduce opioid and other sedative drug consumption. In this systematic review, our primary objective was to summarize available evidence regarding the impact of continuous ketamine infusion on opioid and sedative drug consumption in adult and pediatric critically ill patients. Our secondary objectives included evaluating the effects of ketamine on the duration of MV, ICU, and hospital LOS, level of sedation and pain, adverse events (e.g., intracranial pressure [ICP] elevation, incidence of delirium), and all-cause mortality.

## Methods

### Protocol and Registration

This systematic review was designed, conducted, and reported in accordance with Preferred Reporting Items for Systematic Reviews and Meta-Analysis (PRISMA) guidelines (Appendices Table A1).^10^ The protocol is available in the PROSPERO international prospective registry (PROSPERO 2020 CRD42020173693).

### Eligibility Criteria

A broad search strategy was set to identify studies that compared continuous infusion ketamine versus any nonketamine-containing analgosedation regimens (e.g., opioids, propofol, dexmedetomidine, and benzodiazepines) in critically ill patients. Randomized controlled trials (RCTs), quasi-experimental studies, and observational cohort studies were eligible; cross-over designs were excluded. Pre-post study designs in which comparisons were made within participants were also excluded due to a time-dependent bias in the critically ill patients where patient status may deteriorate with time making postintervention results difficult to compare with those preintervention. Studies were not limited by language, geographic location, year of publication, or subject age. Only published studies were included. Abstracts and ongoing studies were qualitatively reviewed and excluded from the main analysis.

All eligible studies must have included our primary outcome of interest: daily opioid and/or sedative consumption during ICU or hospital stay. Secondary outcomes were the duration of MV, ICU LOS, hospital LOS, level of sedation, adverse events (e.g., ICP elevation, incidence of delirium), and all-cause mortality.

### Information Sources and Search

The following databases were searched from inception until November 19, 2021: MEDLINE (Ovid), EMBASE (Ovid), CINAHL, Cochrane Central Database of Controlled Trials and Systematic Reviews (CDSR), and ClinicalTrials.gov. The search strategy was designed by an experienced medical librarian (CDC) and included concepts for study population, drug, and indication using terms and keywords derived from scoping search and expertise in the subject field (Appendices Table A2 presents the MEDLINE search strategy, including search terms and relevant Medical Subject Headings [MeSH]).

### Study Selection, Data Collection Process, and Data Items

Two reviewers (K.C. and D.R.W.) independently screened titles and abstracts of identified studies for inclusion based on above eligibility criteria using Covidence software. Google Translate was used when screening non-English articles. A third reviewer (L.D.B.) resolved discrepancies or undecided cases. Full text was obtained for agreed upon studies and independently screened by two reviewers (K.C. and D.R.W.). Reference lists of relevant studies were screened by to identify other relevant studies (K.C.).

Data were abstracted using a standardized form in Excel by two independent reviewers (K.C. and C.T.). Study characteristics, including author, country, publication year, population, intervention, comparator, randomization, blinding, study drug protocol, funding, ICU type, study design, sample size, sample demographics, follow-up period, inclusion and exclusion criteria, and outcomes, were collected. While study authors were contacted for missing data, no additional information was acquired.

### Risk of Bias

Two reviewers (K.C. and C.T.) independently assessed the risk of bias for each included study. The Cochrane Collaboration tool for Assessing Risk of Bias 1 (RoB) was used for RCTs and the Risk of Bias In Nonrandomized Studies—of Interventions (ROBINS-I) tool for cohort studies.^11,12^ Discrepancies were mediated by a third reviewer (D.R.W.).

### Summary Measures and Synthesis of Results

When pooling of outcome data was appropriate, RevMan software was used to conduct meta-analyses (Review Manager [RevMan] Version 5.4, The Cochrane Collaboration, 2020). As per the protocol, only RCTs were combined in the main meta-analysis due to a sufficient number identified. Observational studies were retained for a separate analysis and qualitative purposes. All opioids were converted into morphine equivalents (MEQ),^13^ whereas sedatives (benzodiazepines) were included when convertible to midazolam equivalents. Where conversion of opioid doses was required and weight was not reported, a standard of 75 kg average patient weight was assumed. Cumulative opioid doses were divided by measurement time point to estimate dose per kg per hour. All studies reporting benzodiazepine consumption used midazolam, and therefore no conversion was necessary. Statistical heterogeneity was measured using the I^2^ statistic.

Mean difference (MD) summarized the primary outcome (opioid and sedative consumption) with 95% confidence intervals (CI). Study data were pooled for meta-analyses in a random effects model where outcome measures were comparable. Meta-analyses were performed for morphine and midazolam equivalents consumption,^13^ duration of MV, ICU LOS, hospital LOS, ICP, and mortality. All outcomes were summarized using an MD with the exception of mortality using odd ratios with 95% CI. Due to heterogeneity in reporting of mortality, we used the last mortality data reported (hospital mortality^4,14-16^ and ICU mortality^17^). RCTs reporting mortality over a shortened defined period (≤5 days) were excluded from this analysis.^16,18-23^ When needed and to enable meta-analysis, means and standard deviations were estimated using medians and interquartile ranges as previously described.^24^ Due to the small number of included studies, an Egger’s test was not performed to assess publication bias.^25^

### Risk of Bias Across Studies

Within study, selective reporting of outcomes was examined by comparing the *a priori* outcomes listed in the *Methods* section with those reported in the *Results* section. The GRADE framework (Grading of Recommendations, Assessment, Development and Evaluations) was used to rate the quality of evidence^26^ for each pooled outcome undergoing meta-analysis by two reviewers (L.D.B./D.R.W.). An overall quality rating is applied (very low, low, moderate, or high) to describe the certainty in the evidence.

## Results

### Study Selection

**Figure 1.**
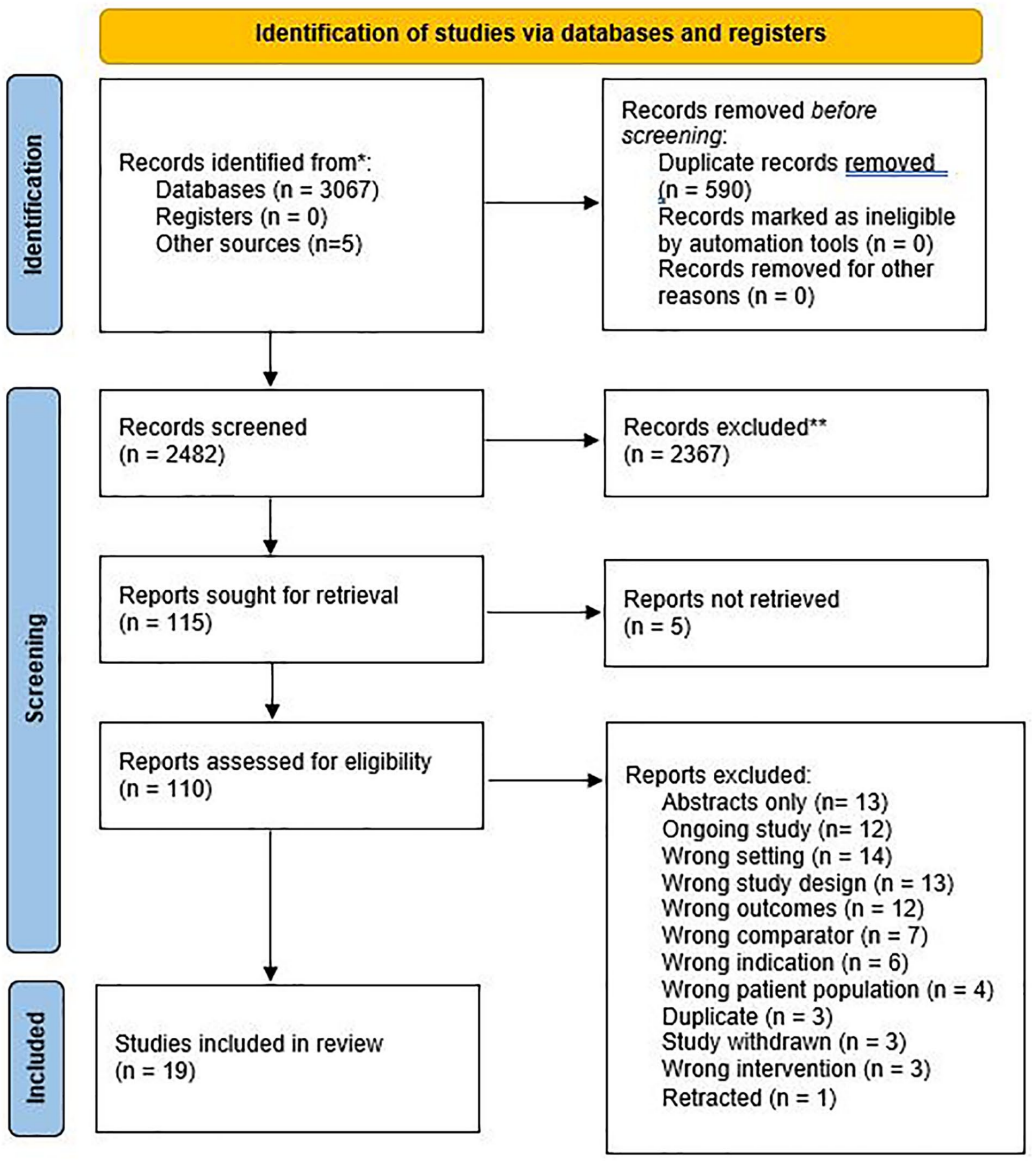
PRISMA 2020 flow diagram for new systematic reviews. Abbreviation: PRISMA, Preferred Reporting Items for Systematic Reviews and Meta-Analysis.

The search yielded 3067 potentially relevant citations of which after removing duplicates and screening titles and abstracts, 110 citations were reviewed in full (Figure 1). However, 19 studies met the inclusion criteria: 13 RCTs^4,14,15,17-23,27,28^ and 6 observational cohort studies.^29-33^

### Study Characteristics

The characteristics of the included studies and key results are summarized in Tables 1 and 2, respectively. Critically ill patient populations, ketamine regimens, and dosing were heterogeneous (Table 1).

**Table 1.** Characteristics of Included Studies Grouped by Date Within Interventions.

Author, year	Setting	Study type	N	Follow-up period	Intervention	Control	Inclusion criteria
Experimental studies							
Kolenda et al^17^	Germany, mixed ICU	RCT, unblinded	24	Up to 14 days of sedation	Ketamine + midazolam	Fentanyl + midazolam	Patients with moderate or severe head injury, age 16-72 years, analgosedation ≥ 3 days
Christ et al^18^	Austria, mixed ICU	RCT, blinding unclear	25	24 hours	Ketamine + midazolam	Sufentanil + midazolam	MV patients with catecholamine-dependent heart failure
Kim et al^28^	Korea, mixed ICU	RCT, blinding unclear	38	24 hours	Ketamine + midazolam	Morphine + midazolam	MV patients
Bourgoin et al^22^	France, ICU in trauma center	RCT, double-blind	25	First 4 days of sedation	Ketamine + midazolam	Sufentanil + midazolam	MV patients with TBI and CT scan indicating significant risk of increased ICP, age 16-75 years
Bourgoin et al^27^	France, ICU in trauma center	RCT, blinding unclear	30	24 hours of target sedation, then plasma concentration doubled for 15 minutes	Ketamine + midazolam	Sufentanil + midazolam	Patients with severe TBI requiring ICP monitoring, age 18-75 years
Schmittner et al^19^	Germany, mixed ICU	RCT, blinding unclear	24	5 days	S(+)-Ketamine + methohexitone	Fentanyl + methohexitone	Patients with severe TBI or aSAH and ICU ventilation > 12 hours, age > 18 years
Quisilema-Cadena et al^15^	Cuba, mixed ICU	RCT, unblinded	18	Sep 2014-May 2015 (until discharge)	Ketamine + midazolam	Morphine + midazolam	MV patients
Guillou et al^20^	France, surgical ICU	RCT, double-blind	93	48 hours	Ketamine + morphine PCA	Normal saline + morphine PCA	Ventilated patients postabdominal surgery, age > 18 years
Minoshima et al^21^	Japan, mixed ICU	RCT, double-blind	36	48 hours	Ketamine + morphine PCA	Normal saline + morphine PCA	Patients undergoing posterior correction surgery for adolescent idiopathic scoliosis, age 10-19 years
Anwar et al^23^	The United Kingdom, cardiac ICU	RCT, double-blind	150	48 hours	Ketamine + pregabalin + morphine PCA	Placebo + pregabalin + morphine PCA	Patients postelective cardiac surgery through sternotomy, age 18-80 years
Dzierba et al^14^	Dzierba, medical ICU	RCT, unblinded	20	Until discharge, death or 7 days	Ketamine + fentanyl/hydromorphone + midazolam	Fentanyl/hydromorphone + midazolam	MV patients receiving ECMO for severe ARDS and requiring deep sedation, age ≥ 18 years
Perbet et al^4^	France, mixed ICU	RCT, double-blind	162	90 days	Ketamine + remifentanil + midazolam/propofol	Normal saline + remifentanil + midazolam/propofol	MV patients requiring MV > 24 hours, age ≥ 18 years
Amer et al^16^	Saudi Arabia, medical, surgical, and transplant ICUs	RCT, unblinded	83	28 days	Ketamine + fentanyl + propofol	Fentanyl + propofol	Patients requiring MV > 24 hours, age ≥ 18 years
Observational studies							
Von der Brelie et al^30^	Germany, mixed ICU	Retrospective cohort	65	June 2010-Dec 2013 (until discharge)	Ketamine in sedative regimen	No ketamine in sedative regimen	Patients with aSAH requiring sedation
Reese et al^31^	The United States, ICU at tertiary center	Retrospective control group and prospective ketamine group	46	Jan 2012-April 2015 (until discharge)	Ketamine as primary sedative for 48 hours or less	No ketamine use (historical control)	MV patients with septic shock requiring sedation, age 18-89 years
							Historical control group: 2010-2011
							Prospective cohort study: 2012-2015
Park et al^29^	Korea, tertiary PICU	Retrospective cohort	240	Jan 2015-Dec 2017 (until discharge)	Ketamine (mostly add-on) + sedative (mostly opioids)	Sedative (mostly opioids)	Patients sedated for ≥ 24 hours
Shurtleff et al^32^	The United States, ICU at tertiary center	Retrospective cohort	79	Nov 2015-Apr 2017 (up to 12 days)	Ketamine in sedative regimen for at least 6 hours	Propofol	Patients receiving analgosedation for ≥ 6 hours, age ≥ 18 years
Jaeger et al^33^	The United States, medical ICU	Retrospective cohort	172	Jan 2013-Dec 2018	Ketamine in sedative regimen	Non-ketamine sedation	MV patients (≥ 24 hours) receiving continuous infusion sedation (≥ 6 hours), age ≥ 18 years
Wu et al^34^	Netherlands, medical-surgical ICU	Post hoc subgroup of prospective cohort	925	Jul 2016-Feb 2020	Ketamine in sedative regimen	Non-ketamine sedation	Patients receiving analgosedation and expected to survive ≥ 48 hours

Abbreviations: ARDS, acute respiratory distress syndrome; aSAH, aneurysmal subarachnoid hemorrhage; ECMO, extracorporeal membrane oxygenation; GCS, Glasgow Coma Score; h, hours; ICP, intracranial pressure; ICU, intensive care unit; MV, mechanical ventilation; PCA, patient-controlled analgesia; PICU, pediatric intensive care unit; RCT, randomized controlled trial; TBI, traumatic brain injury.

**Table 2.** Key Results of Included Studies Grouped by Date Within Interventions.

Author, year	Ketamine dosing	Main outcomes	Key results
Experimental studies			
Kolenda et al^17^	Ketamine 65 mg kg^−1^ day^−1^ (2.7 mg kg^−1^ h^−1^) adjusted to clinical requirements	Recovery of motor response, sedation/analgesia, sedative and opioid consumption, MAP, ICP	Median midazolam consumption was similar in the ketamine and fentanyl groups (11.1 vs 10.7 mg kg^−1^ day^−1^). Additional sedatives were required in 2 ketamine patients and 4 fentanyl patients. However, 3 patients in the ketamine group and 1 in the fentanyl group died during follow-up. Incidence of persistent ICP (>25 mmHg) was similar in the two groups.
Christ et al^18^	Ketamine titrated to RSS = 5	Hemodynamic parameters, catecholamine requirements, midazolam consumption	Mean midazolam dose was not significantly different in the ketamine versus sufentanil groups (0.12 vs 0.15 mg kg^−1^ h^−1^). Comparable sedation was achieved in both groups using ketamine mean 2.5 mg kg^−1^ h^−1^ and sufentanil 0.88 µg kg^−1^ h^−1^.
Kim et al^28^	Unclear	Hemodynamic changes	Mean midazolam consumption was nonsignificantly higher in the ketamine versus morphine group (52.1 vs 46.7 mg day^−1^).
Bourgoin et al^22^	Ketamine 50 µg kg^−1^ min^−1^ (3 mg kg^−1^ h^−1^), adjusted to ICP and CPP levels	ICP, CPP	Mean midazolam dose was similar in the ketamine and sufentanil groups (1.64 vs 1.63 µg kg^−1^ min^−1^). After infusions were stopped, improvement in GCS score was faster in the nonketamine group. However, GCS score was similar at patient recovery. Four patients in the ketamine group and 3 in the comparator died during the study. Mean daily ICP and CPP values were similar.
Bourgoin et al^27^	Infusion to plasma concentration 1.0 µg mL^−1^, adjusted to pain scores	ICP, CPP, MAP, and drug plasma concentrations after increasing dose	Mean BIS was 74 versus 65 in the ketamine and sufentanil groups (nonsignificant). Mean ICP values were similar.
Schmittner et al^19^	Ketamine 0.5 mg kg^−1^ bolus, then titrated to target sedation (maximum 2 mg kg^−1^ h^−1^)	MAP, CVP, ICP, and CPP	Mean sedation levels measured by BIS and ICP were similar from days 1 to 5. Persistent ICP (>20 mmHg) was reported in 8 ketamine and 6 fentanyl patients.
Quisilema-Cadena et al^15^	Ketamine 0.3 mg kg^−1^ bolus, then 0.05-0.4 mg kg^−1^ h^−1^ increased by 0.05 mg kg^−1^ h^−1^ q60 min until adequate sedation achieved	Time to extubation, RASS	Mean midazolam daily dose was not significantly different between the ketamine and morphine group (0.1 vs 0.1 mg kg^−1^ day^−1^).
	Median ketamine dose 0.6 mg kg^−1^ day^−1^ (0.025 mg kg^−1^ h^−1^)		
Guillou et al^20^	Ketamine 0.5 mg kg^−1^ bolus, then 2 µg kg^−1^ min^−1^ (0.12 mg kg^−1^ h^−1^) during the first 24 hours, then 1 µg kg^−1^ min^−1^ (0.06 mg kg^−1^ h^−1^)	VAS pain scores, opioid consumption	Mean morphine consumption was significantly lower in the ketamine versus placebo group (58 vs 80 mg at 48 hours).
Minoshima et al^21^	Ketamine 0.5 mg kg^−1^ bolus during surgery, then 2 µg kg^−1^ min^−1^ (0.12 mg kg^−1^ h^−1^) postoperatively for 48 hours	Morphine consumption, pain and sedation scores	Cumulative mean morphine consumption was significantly lower in the ketamine versus placebo group at 24 hours (0.59 vs 0.75 mg kg^−1^) and 48 hours (0.89 vs 1.16 mg kg^−1^). Sedation levels were similar at 24 hours after ICU arrival. Hospital LOS were also similar. No delirium nor psychomimetic effects were observed in either group.
Anwar et al^23^	Ketamine 0.1 mg kg^−1^ h^−1^ for 48 hours postoperatively	Pain at 3 and 6 months, acute pain, opioid use, and analgesic requirements	Median morphine consumption was significantly lower in the ketamine group by 4 mg/day. Sedation scores, ICU LOS, and hospital LOS were similar.
Dzierba et al^14^	Ketamine 40 mg bolus, then 5 µg kg^−1^ min^−1^ (0.3 mg kg^−1^ h^−1^)	Opioid and sedative consumption, renal replacement therapy, and delirium	Median midazolam consumption was not significantly different between ketamine and nonketamine groups (8 vs 6 mg day^−1^). Median fentanyl consumption was also similar (6 vs 5 mg day^−1^). The duration of MV, ICU LOS, and hospital LOS was similar. Mortality was equal among the two groups. Delirium was present in 7 patients in the ketamine group and 9 of the control group on the day of waking.
Perbet et al^4^	Ketamine 3.3 µg kg^−1^ min^−1^ (0.2 mg kg^−1^ h^−1^)	Daily opiate consumption, delirium, opioid consumption, and ventilation days	Both mean midazolam and propofol consumption were similar between the ketamine and placebo groups (midazolam 1.4 vs 1.6 mg kg^−1^ day^−1^; propofol 31 vs 35 mg kg^−1^ day^−1^). Mean reimfentanil consumption was not significantly different (7.9 µg kg^−1^ h^−1^ vs 9.3 µg kg^−1^ h^−1^). Median duration of MV and ICU stay was similar. ICU mortality was 35% (n = 28) and 43% (n = 35); hospital mortality was 39% (n = 31) and 45% (n = 37).
			Incidence of delirium was significantly lower in the ketamine group (21% vs 37%, *P* = 0.03). When present, delirium was also significantly shorter in the ketamine group (mean 2.8 vs 5.3 days, *P* = 0.005).
Amer et al^16^	Ketamine 1-2 µg kg^−1^ min^−1^ (0.06-0.12 mg kg^−1^ h^−1^)	Consent, recruitment, and protocol adherence rate	Both median propofol and fentanyl consumption were similar between ketamine and placebo groups (propofol 28.0 vs 28.4 mg kg^−1^ at 48 hours; fentanyl 69.6 vs 63.5 µg kg^−1^ at 48 hours). Percent of patients at 24-hour goal RASS was 67.5% in the ketamine group and 52.4% in the control group (*P* = 0.24) (at 48 hours, 73.5 and 66.7%, *P* = 0.70)
Observational studies			
Von der Brelie et al^30^	Ketamine up to a maximum of 500 mg h^−1^ (~6.7 mg kg^−1^ h^−1^)	ICP and vasopressor consumption	None of the patients in the ketamine group experienced a critical increase in ICP after administration of ketamine. Mortality and adverse events were similar in the two groups.
Reese et al^31^	Ketamine 1-2 mg kg^−1^ bolus, then 5 µg kg^−1^ min^−1^ (0.3 mg kg^−1^ h^−1^) with a titration of 2 µg kg^−1^ min^−1^ q30 min to adequate sedation	Vasopressor consumption, ketamine consumption, other sedative and analgesic use, and MV days	Total benzodiazepine dose was significantly lower at 24 hours in the ketamine group (10 vs 42 mg), and nonsignificantly at 48 hours (26 vs 75 mg). Cumulative fentanyl dose at 48 hours was significantly lower in the ketamine group (429 vs 2235 µg).
Park et al^29^	Median ketamine infusion rate of 8.1 µg kg^−1^ min^−1^ (0.49 mg kg^−1^ h^−1^)	BP, HR, RR, vasogenic medications, mortality, sedation, and pain scores	In total, 64 patients in the ketamine group vs 120 patients in the nonketamine group received fentanyl. The duration of MV was significantly shorter in the ketamine group (median 17.0 vs 7.5 days). Hospital and ICU LOS were also significantly lower in the ketamine group.
Shurtleff et al^32^	Ketamine 5 µg kg^−1^ min^−1^, titrated by 5 µg kg^−1^ min^−1^ q5 min up to max 25 µg kg^−1^ min^−1^; median infusion dose was 7 µg kg^−1^ min^−1^ (0.42 mg kg^−1^ h^−1^)	Days without delirium or coma	Ketamine was mostly used in patients who failed first-line sedation regimens. No significant difference in delirium was detected. Total midazolam and fentanyl consumption were not significantly different between groups. Median ICU (15 vs 12 days) and hospital LOS (11 vs 8 days) were longer in the ketamine groups.
Jaeger et al^33^	Median ketamine infusion rate of 7.9 µg kg^−1^ min^−1^ (0.47 mg kg^−1^ h^−1^)	% of RASS and pain scores at goal, sedative, and vasopressor consumption	Ketamine was mostly used in patients who failed first-line sedation regimens. No significant difference was found in midazolam nor fentanyl consumption. ICU LOS was longer in the ketamine group (8.8 vs 5.2 days).
Wu et al^34^	0.50 mg kg^−1^ h^−1^ in the delirium incidence group and 0.12 mg kg^−1^ h^−1^ in the no delirium group	Incident ICU delirium	Ketamine use was greater in patients with delirium (16% vs 0.7%, *P* < 0.01). Note that a greater proportion of ketamine patients were urgent surgical admissions (54% vs 34%, *P* = 0.01) and received average IV MEQ dose ≥ 10 mg day^−1^ (91% vs 75%, *P* = 0.02).

Abbreviations: BIS, bispectral index; BP, blood pressure; CPP, cerebral perfusion pressure; CVP, central venous pressure; GCS, Glasgow Coma Scale; HR, heart rate; ICP, intracranial pressure; ICU, intensive care unit; IV, intravenous; LOS, length of stay; MAP, mean arterial pressure; MV, mechanical ventilation; RASS, Richmond Agitation-Sedation Scale; RR, respiratory rate; RSS, Ramsay Sedation Scale; VAS, visual analogue scale.

The included RCTs were generally small (range N = 18-162) compared with cohort studies (N = 46-925 subjects). Seven RCTs compared the use of ketamine in combination with a sedative (6 midazolam, 1 methohexitone) to an opioid with sedative in adult head-injured patients^17,19,22,27^ or MV patients.^15,18,28^ Three RCTs compared ketamine in combination with morphine patient-controlled analgesia (PCA) to placebo with morphine PCA following major surgery (adolescents^21^ or adults^20,23^). Three RCTs compared ketamine in combination with both an opioid and sedative to the same regimen without ketamine in MV adults.^4,14,16^ The sedation regimens in the 6 cohort studies were less clear, as patients in the control group received usual care. Subjects in the comparator group generally received ketamine as an add-on, where traditional sedatives or analgesics were not sufficient to maintain adequate sedation. Patient demographics also varied among the cohort studies, with inclusion criteria ranging from any patients who received prolonged sedation (≥ 6 hours) to patients receiving MV, or with specific diagnoses, such as subarachnoid hemorrhage or septic shock. RCTs were conducted in France, Germany, Austria, Korea, Cuba, Japan, the United Kingdom, the United States, and Saudi Arabia. Observational studies were conducted in Korea, Germany, the United States, and the Netherlands.

Seven RCTs were excluded from pooling data for opioid consumption due to lack of reporting as ketamine was used to replace an opioid in the regimen.^15,17-19,22,27,28^ Seven RCTs were excluded from pooling data for midazolam consumption: (1) two were missing appropriate data,^17,27^ (2) four did not include midazolam in the analgosedative regimen,^19-21,23^ and (3) one only used midazolam as an alternative to propofol (eg. allergy).^16^ Here, 7 RCTs did not report length of MV due to a non-MV study population^17,19-23,27^ and 3 due to a lack of reporting on the outcome.^15,18,28^ Despite all 13 RCTs taking place in the ICU population, 7 did not report ICU LOS as an outcome^17-21,27,28^ and 1 study only reported on ICU LOS for both treatment groups combined.^15^ Similarly, 8 of the 13 RCTs did not report hospital LOS.^4,15,17-20,22,27^

Ketamine dosing strategies varied widely among the studies. Some studies (n = 6, 32%) employed a bolus of ketamine prior to continuous infusion.^14,15,19-21,31^ Continuous infusions ranged from 0.025^15^ to 3.0 mg kg^−1^ h^−122^ with the lower end of the dose range generally used for PCA.

### Risk of Bias

**Figure A1.**
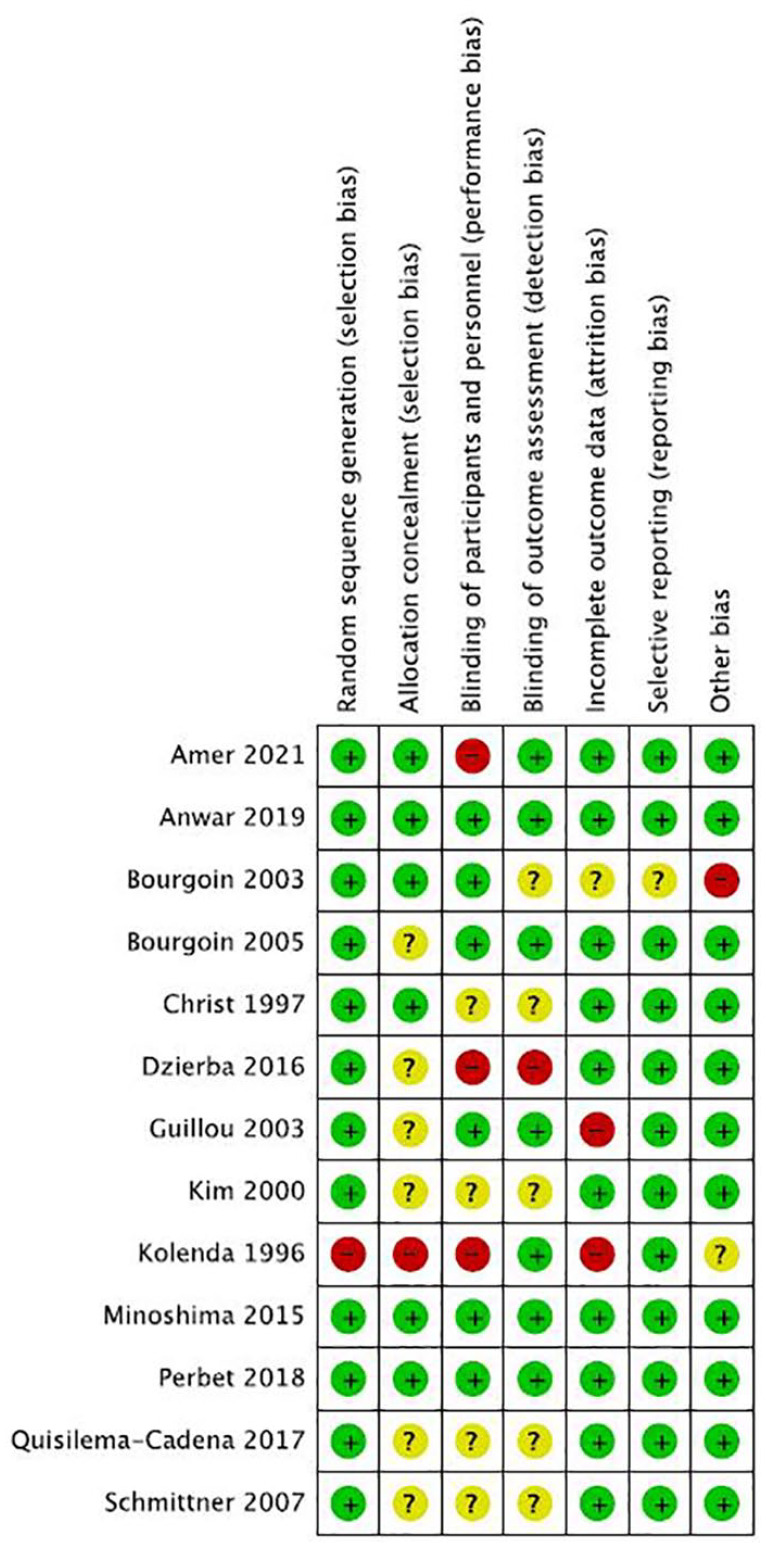
Bias assessment of included RCTs using the Cochrane RoB 1 tool (n = 13). Abbreviations: RCT, randomized controlled trials; RoB 1, risk of bias 1.

**Figure A2.**
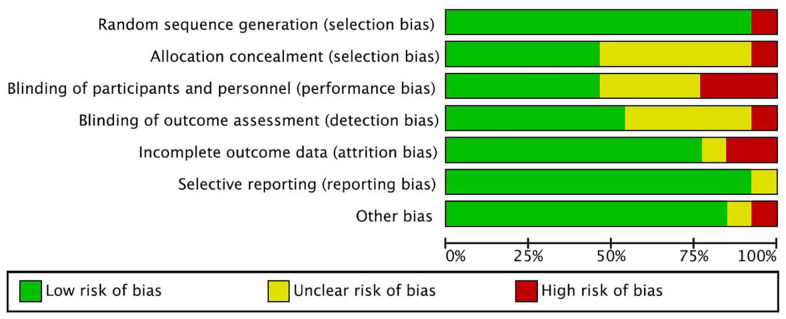
Breakdown of bias of included RCTs using the Cochrane RoB 1 tool (n = 13). Abbreviations: RCT, randomized controlled trials; RoB 1, risk of bias 1.

The risk of bias was scored as high in 5 of the 13 RCTs. Three studies were due to lack of blinding,^14,16,17^ 1 due to exclusion of patients who reached maximum sedative doses,^19^ and 1 due to concerns of missing outcome data, bias in measurement of the outcome, and in selection of the reported results^22^ (Figures A1 and A2). Three studies were assessed with low risk of overall bias due to appropriate RCT design and complete reporting.^4,21,23^ Five studies were noted to have some concerns primarily due to allocation concealment and blinding not explicitly reported.^15,18,19,27,28^

Quality assessment of observational cohort studies using ROBINS-I indicated high risk of bias in all 6 studies. An immortal time bias was noted in observational studies; patients in the treatment arm entered the treatment group at later points during analgosedation as ketamine was typically used as an adjunct when traditional regimens failed to maintain adequate sedation. In addition, a selection bias exists in the retrospective cohort studies whereby ketamine was mainly added as an adjunctive analgosedative when traditional regimens were insufficient.^29,30^

### Synthesis of Results

Heterogeneity across studies was high, and in our main analysis, only data from RCTs were pooled. However, observational studies were pooled for hypothesis generating purposes and guiding future research. Meta-analyses were not performed on the following outcomes due to lack of outcome data or factors that made pooling inappropriate: sedation levels, pain, and adverse events. Outcomes are described qualitatively and in Tables 1 and 2.

#### Opioid consumption

**Figure 2.**
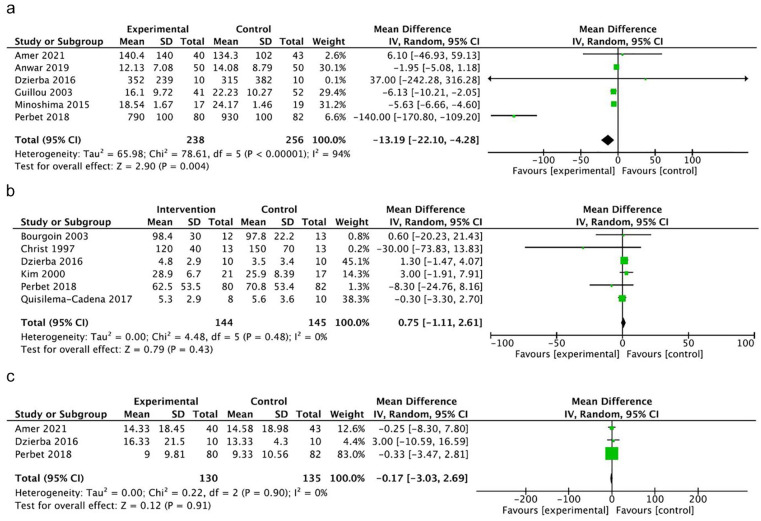
Forest plots of comparison. (a) Mean morphine equivalent dose (ME) (µg kg^−1^ h^−1^). (b) Mean midazolam dose (µg kg^−1^ h^−1^). (c) Mean duration of MV (days). Abbreviations: CI, confidence interval; IV, intravenous; MV, mechanical ventilation.

Overall, 6 RCTs reporting opioid consumption were pooled with 494 participants (Figure 2a).^4,14,16,20,21,23^ Opioid consumption was 13.19 µg kg^−1^ h^−1^ MEQ less (MD, 95% CI −22.10 to −4.28, *P* = 0.004, very low certainty) in the ketamine group. An I^2^ of 94% suggests high heterogeneity across the studies, likely owing to the range of target study populations. Three of the studies enrolled postoperative patients in the ICU receiving morphine PCA (with or without adjunctive continuous infusion ketamine) all with similar dosing regimens.^20,21,23^ Another study focused on patients on MV and ECMO—sample size was small (n = 20) and the study was weighted only 0.1% in the meta-analysis.^14^ Two studies focused on MV patients—one reported a relatively large reduction in opioid consumption^4^ and the other an increase in the ketamine group.^16^

**Figure A3.**
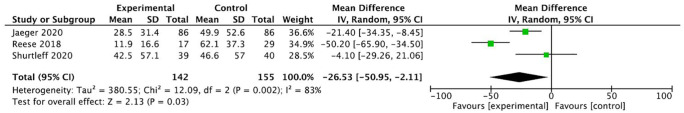
Forest plot comparison of opioid consumption across observational studies. Abbreviations: CI, confidence interval; IV, intravenous.

Pooling of the 3 observational studies reporting adequate data revealed a significant reduction of opioids (MD −26.53 µg kg^−1^ h^−1^ ME, 95% CI −50.95 to −2.11, *P* = 0.03, very low certainty) (Figure A3).^31-33^ This is in alignment with results from meta-analysis of the RCTs.

#### Sedative consumption

All studies reporting benzodiazepine consumption used midazolam.^4,14-16,18,22,28^ One study was excluded from meta-analysis, as midazolam was used only as an alternative to propofol for reasons, such as an allergy.^16^ Meta-analysis of mean midazolam dose across the 6 RCTs with 289 participants^4,14,15,18,22,28^ demonstrated no difference between groups treated with and without ketamine (MD 0.75 mg kg^−1^ h^−1^, 95% CI −1.11 to 2.61, *P* = 0.43, very low certainty) (Figure 2b). An I^2^ of 0% suggests minimal heterogeneity.

#### Duration of MV

Mean duration of MV was reported in 3 RCTs with 265 participants.^4,14,16^ No difference between ketamine and nonketamine groups was identified (MD −0.17 days, 95% CI −3.03 to 2.69, *P* = 0.91, very low certainty) (Figure 2c). An I^2^ of 0% suggests minimal heterogeneity. The duration of MV was significantly longer in one cohort study^29^ and comparable in the remaining where reported,^31,32,34^ but data were not pooled due to bias. Patients typically received ketamine following the failure of first-line regimens in achieving goal analgosedation—therefore, those who received ketamine were more likely to be on MV longer.

#### ICU and hospital LOS

**Figure A4.**
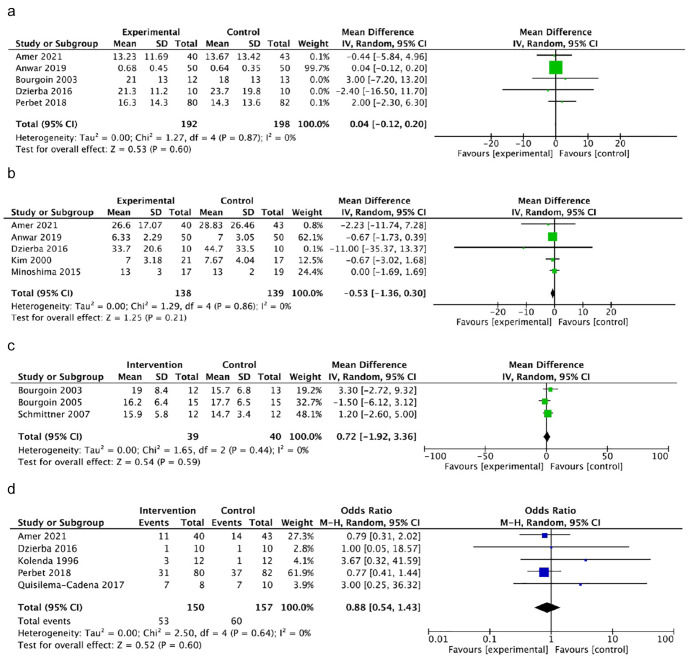
Forest plot of comparison across RCTs: (a) Mean length of ICU stay (days), (b) Mean length of hospital stay (days), (c) Intracranial pressure (ICP, mmHg), and (d) Mortality. Abbreviations: CI, confidence interval; IV, intravenous; ICU, intensive care unit; ICP, intracranial pressure; RCT, randomized controlled trials.

Meta-analysis of mean ICU LOS across 390 patients in 5 studies^4,14,16,22,23^ demonstrated no difference between the ketamine and nonketamine groups (MD 0.04 days, 95% CI −0.12 to 0.20, *P* = 0.60, low certainty) (Figure A4a). An I^2^ of 0% suggests minimal heterogeneity. Similarly, no significant difference in hospital LOS was observed across the 277 patients in 5 studies^14,16,21,23,28^ (MD −0.53 days, 95% CI −1.36 to 0.30, *P* = 0.21, low certainty) (Figure A4b). An I^2^ of 0% suggests minimal heterogeneity. Hospital LOS^29,32^ and ICU LOS^29,32,33^ were longer in several individual cohort studies (*P* < 0.05), but data were not pooled due to bias.

#### Intracranial pressure

ICP elevation was described as an outcome in 3 RCTs of brain injury patients.^19,22,27^ Meta-analysis of 79 participants across the 3 RCTs demonstrated no significant difference with ketamine administration (MD 0.72 mmHg, 95% CI −1.92 to 3.36, *P* = 0.59, low certainty) (Figure A4c). An I^2^ of 0% suggests minimal heterogeneity.

#### Mortality

Mortality was comparable between groups in RCTs.^4,14-17^ Meta-analysis of 307 patients across the 5 RCTs demonstrated no significant difference with ketamine administration (odds ratio 0.88, 95% CI 0.54-1.43, *P* = 0.60, low certainty) (Figure A4d). Cohort studies demonstrated a nonsignificant association of increased mortality in the ketamine group, possibly owing to selection bias whereby ketamine was administered as an adjunctive analgosedative when first-line sedation regimens were inadequate.^29-34^

#### Other outcomes described qualitatively

Qualitative descriptions of outcomes and ketamine doses are further detailed in Table 2. Sedation levels were comparable between treatment groups. In the majority of studies (5 RCTs^4,14-16,21^ and 6 observational studies^29-34^), sedation evaluation used the Richmond Agitation-Sedation Scale (RASS). A nurse was reported to perform these assessments in 3 of the RCTs^4,16,21^ and 3 observational studies,^32-34^ while the remaining were unclear.^14,15,29-31^ Another 4 RCTs used the Ramsay Sedation Scale (RSS)—one measured by a nurse,^19^ another by a blinded observer,^20^ and the remaining unclear.^18,28^ Anwar et al used a sedation score based on a Likert scale,^23^ Bourgoin et al a behavioral pain scale,^27^ and Bourgoin et al clinical judgment during endotracheal suction.^22^ The remaining study did not report assessing sedation.^17^ Of the study designs that allowed comparison of methohexitone and propofol consumption, no difference between ketamine and nonketamine groups was found.^4,16,19^ Sedation results were not pooled as different measures were used across the trials at varying time points.

A variety of tools were used to measure pain (behavioral pain scale,^4,27^ bispectral index,^19^ visual analogue scale,^20^ numerical rating scale,^4,21,23^ a handheld pressure algometer and monofilament von Frey fibers,^23^ critical care pain observation tool,^16,32^ face pain scale or Face-Leg-Activity-Cry-Consolability score,^29^ self-reported pain scores,^33^ nonverbal pain scale^33^). In the majority of studies, the assessor was unidentified, while three reported nurse measurement,^4,21,32^ and one a blinded observer.^20^ Pain scores were comparable between groups where reported (7 RCTs^4,16,19-21,23,27^ and 3 observational studies^29,32,33^). However, in the observational study by Shurtleff et al, a greater percentage of patients in the ketamine group achieved target pain scores (99% vs 91%, *P* = 0.04).^32^

All but 3 studies^18,28,29^ reported the occurrence of adverse events. Reports of adverse events were generally similar between ketamine and morphine groups. Two studies reported hypotension was experienced in a greater proportion of the nonketamine group (ketamine n = 2, 9% vs morphine group n = 5, 29%;^28^ n = 2, 25% vs n = 7, 70%^15^). One RCT and one cohort study reported greater vasopressor use in the nonketamine groups (ketamine n = 6, 50% vs sufentanil n = 8, 62%;^22^ ketamine n = 6, 35% vs n = 18, 62% nonketamine,^31^ *P* > 0.05). In the study of postoperative cardiac patients, diplopia occurred more often in the ketamine plus pregabalin group versus pregabalin alone (number needed to harm 4.5 vs 6.3).^23^ A significantly lower incidence of delirium was also observed in ketamine groups across several studies. Perbet et al reported a significant reduction of delirium in the ketamine group (n = 17, 21% versus n = 30, 37%, *P* = 0.03).^4^ This observation is echoed with a nonsignificant reduced incidence in the RCT of ECMO patients (n = 7, 70% vs n = 9, 90%)^14^ and retrospective cohort of general ICU patients (n = 29, 74% vs n = 34, 85%).^32^ In contrast, an observational study reported ketamine use was more likely in patients who experienced delirium versus those who did not (n = 54, 16% versus n = 4, 0.7%, *P* < 0.01).^16^

## Discussion

The results of our systematic review and meta-analysis suggest ketamine may have benefits as an analgosedative in the ICU. Ketamine was found to decrease opioid consumption in the ICU, with no evidence of effect on sedation consumption.

Despite the high frequency of analgosedation in the ICU, practice patterns indicate choice, combination, and dosing of conventional sedatives and opioids remain highly variable.^35^ Opioids are limited by tolerance, hyperalgesia, increased risk of withdrawal, and propensity to reduce blood pressure^4^; propofol by hypotension and hemodynamic instability^7^; benzodiazepines by risk of respiratory and cardiovascular depression, delirium, and unintended oversedation from drug accumulation^1^; and dexmedetomidine by hypotension, bradycardia, and cost.^1,8^ Choice of sedative and regimens is heavily dependent on local practices and clinical judgment. Ketamine has been a subject of interest in several studies and systematic reviews^9,36^ for its distinct profile and positive hemodynamic effects. To our knowledge, the only other meta-analysis of adjunctive ketamine for analgosedation in critically ill patients was conducted by Manasco et al.^36^ The meta-analysis revealed that ketamine was associated with a reduction in propofol infusion rate but had no impact on fentanyl or midazolam. The capture of studies in our meta-analysis, which excluded pre-post study designs, did not provide sufficient data to analyze propofol use. Similarly, we report no difference in midazolam mean dose. However, in contrast to our findings, Manasco et al did not find a significant reduction in fentanyl (−21.5 µg h^−1^, *P* = 0.11).^36^ This may be owing to several differences: (1) a focus on MV patients where our review included non-MV patients receiving morphine PCAs; (2) analyzing only observational studies (limited by studies using fentanyl) where our analysis included RCTs (opioids converted to MEQ); and (3) inclusion of pre-post study designs which may have introduced a bias where patients received ketamine later in their ICU stay along with escalation of opioids doses. Together, the results suggest a potential opioid-sparing effect of ketamine that may be further elucidated through more RCTs.

While the pooled opioid-sparing effect of ketamine was small, this may be partly explained by the small sample size and identified heterogeneity. Daily cumulative doses of morphine using a PCA for postoperative analgosedation are relatively low compared with opioid administration in MV ICU populations. Heavier weighting of these postoperative PCA studies^20,21,23^ heavily impacted the results by reducing overall MD in opioid consumption. Our review also sought to detect any signal of adverse effects in critically ill populations at the ketamine doses used for analgosedation. While all studies generally reported comparable or null psychomimetic effects, results of delirium were inconsistent.^4,34^

Strengths of this systematic review include an extensive search, broad inclusion criteria, meta-analysis limited to RCTs, and inclusion of new studies^16,34^ not captured in previous reviews. However, results of the meta-analysis should be interpreted with caution due to the heterogeneous nature of the ICU patient populations identified in the studies, ketamine dosing (timing and strength), outcome measures, and various paired drug combinations (sedatives or opioids through infusion or PCA in combination with ketamine). While we recognize the limitations of combining various ICU populations in analysis (e.g. ventilated and nonventilated), all trials represented critically ill ICU patients in pain and increase the generalizability of the findings. By including non-MV patients, likely to have reduced opioid exposure, our results are biased toward the null. Additionally, the available literature does not permit us to determine the effect of ketamine alone. Further limitations include a high or moderate risk of bias due to study structure and reporting in 11 of the 14 RCTs^14-22,27,28^ and all observational studies included.^29-34^ The use of ketamine in observational studies only when first-line sedatives were inadequate to achieve goal sedation creates an immortal time bias and selection bias. Patients received ketamine at a later point during their stay following failure of first-line regimens, creating a bias toward patients with longer LOS in the ketamine group. Taking this into consideration along with the number of RCTs identified, observational studies were excluded from meta-analysis. Additionally, 6 of the RCTs had relatively short follow-up periods (≤ 48 h).^18,20,21,23,27,28^ However, 10 of the 19 RCTs and cohort studies were small (N ≤ 50).^14,15,17-19,21,22,27,28,31^ Small sample sizes may have underpowered studies to detect adverse effects or a stronger opioid-sparing effect.

Additional RCTs exploring ketamine as an adjunct analgosedation agent to reduce opioid requirements would be valuable. Its potential benefit in reducing iatrogenic drug withdrawal and discharge on addictive substances warrants further study in well-designed trials, employing adequate randomization, blinding (ICU staff and data collection) and powered to detect a difference. Treatment groups using a placebo comparator and controlled dosing regimens of adjunctive ketamine and opioid would allow more definitive elucidation of ketamine as an opioid-sparing agent. Additionally, sparsely reported psychomimetic effects and conflicting results of delirium underline the need for better understanding of potential adverse effects from future trials.

## Relevance to Patient Care and Clinical Practice

A current opioid crisis in developed countries is highlighted by the wave of opioid-involved overdoses presenting to emergency departments and overdose-related deaths. This has implications for health care professionals in acute care settings as excessive prescribing during hospitalization and discharge may lead to chronic use.^5,37^ Limiting opioid overprescribing while minimizing undertreatment of pain is a delicate balance, and the evidence base for alternatives and adjuncts, such as ketamine, is limited.

Results of our meta-analysis suggest ketamine as an adjunct analgosedative has the potential to limit opioid exposure, presenting an additional alternative to traditional regimens that would benefit from further study.

## Conclusion

Our findings suggest ketamine in critically ill patients has the potential to have an opioid-sparing effect in postoperative and MV patients in the ICU. While small, the potential for opioid dose reduction warrants further investigation. Further understanding of agents capable of offering analgesia without extended opioid exposure is particularly critical amidst the opioid crisis experienced in many developed nations.

## Appendix

**Table A1.** Preferred Reporting Items for Systematic Reviews and Meta-Analysis (PRISMA) Guidelines.

Section and topic	Item #	Checklist item	Location where item is reported
**TITLE**			
Title	1	Identify the report as a systematic review.	Title
**ABSTRACT**			
Abstract	2	See the PRISMA 2020 for Abstracts checklist.	Abstract
**INTRODUCTION**			
Rationale	3	Describe the rationale for the review in the context of existing knowledge.	Introduction
Objectives	4	Provide an explicit statement of the objective(s) or question(s) the review addresses.	Introduction
**METHODS**			
Eligibility criteria	5	Specify the inclusion and exclusion criteria for the review and how studies were grouped for the syntheses.	Methods
Information sources	6	Specify all databases, registers, websites, organizations, reference lists, and other sources searched or consulted to identify studies. Specify the date when each source was last searched or consulted.	Methods
Search strategy	7	Present the full search strategies for all databases, registers, and websites, including any filters and limits used.	Methods
Selection process	8	Specify the methods used to decide whether a study met the inclusion criteria of the review, including how many reviewers screened each record and each report retrieved, whether they worked independently, and if applicable, details of automation tools used in the process.	Methods
Data collection process	9	Specify the methods used to collect data from reports, including how many reviewers collected data from each report, whether they worked independently, any processes for obtaining or confirming data from study investigators, and if applicable, details of automation tools used in the process.	Methods
Data items	10a	List and define all outcomes for which data were sought. Specify whether all results that were compatible with each outcome domain in each study were sought (e.g. for all measures, time points, analyses), and if not, the methods used to decide which results to collect.	Methods
	10b	List and define all other variables for which data were sought (e.g. participant and intervention characteristics, funding sources). Describe any assumptions made about any missing or unclear information.	Methods
Study risk of bias assessment	11	Specify the methods used to assess risk of bias in the included studies, including details of the tool(s) used, how many reviewers assessed each study and whether they worked independently, and if applicable, details of automation tools used in the process.	Methods
Effect measures	12	Specify for each outcome the effect measure(s) (e.g. risk ratio, MD) used in the synthesis or presentation of results.	Methods
Synthesis methods	13a	Describe the processes used to decide which studies were eligible for each synthesis (e.g. tabulating the study intervention characteristics and comparing against the planned groups for each synthesis [item #5]).	Methods
	13b	Describe any methods required to prepare the data for presentation or synthesis, such as handling of missing summary statistics, or data conversions.	Methods
	13c	Describe any methods used to tabulate or visually display results of individual studies and syntheses.	Methods
	13d	Describe any methods used to synthesize results and provide a rationale for the choice(s). If meta-analysis was performed, describe the model(s), method(s) to identify the presence and extent of statistical heterogeneity, and software package(s) used.	Methods
	13e	Describe any methods used to explore possible causes of heterogeneity among study results (e.g. subgroup analysis, meta-regression).	NA
	13f	Describe any sensitivity analyses conducted to assess robustness of the synthesized results.	NA
Reporting bias assessment	14	Describe any methods used to assess risk of bias due to missing results in a synthesis (arising from reporting biases).	Methods
Certainty assessment	15	Describe any methods used to assess certainty (or confidence) in the body of evidence for an outcome.	Methods
**RESULTS**			
Study selection	16a	Describe the results of the search and selection process, from the number of records identified in the search to the number of studies included in the review, ideally using a flow diagram.	Results
	16b	Cite studies that might appear to meet the inclusion criteria, but which were excluded, and explain why they were excluded.	Results
Study characteristics	17	Cite each included study and present its characteristics.	Results
Risk of bias in studies	18	Present assessments of risk of bias for each included study.	Results
Results of individual studies	19	For all outcomes, present, for each study: (a) summary statistics for each group (where appropriate) and (b) an effect estimate and its precision (e.g. confidence/credible interval), ideally using structured tables or plots.	Results
Results of syntheses	20a	For each synthesis, briefly summarize the characteristics and risk of bias among contributing studies.	Results
	20b	Present results of all statistical syntheses conducted. If meta-analysis was done, present for each the summary estimate and its precision (e.g. confidence/credible interval) and measures of statistical heterogeneity. If comparing groups, describe the direction of the effect.	Results
	20c	Present results of all investigations of possible causes of heterogeneity among study results.	Results
	20d	Present results of all sensitivity analyses conducted to assess the robustness of the synthesized results.	Results
Reporting biases	21	Present assessments of risk of bias due to missing results (arising from reporting biases) for each synthesis assessed.	Results Figures A1 and A2
Certainty of evidence	22	Present assessments of certainty (or confidence) in the body of evidence for each outcome assessed.	Results (GRADE assessment)
**DISCUSSION**			
Discussion	23a	Provide a general interpretation of the results in the context of other evidence.	Discussion
	23b	Discuss any limitations of the evidence included in the review.	Discussion
	23c	Discuss any limitations of the review processes used.	Discussion
	23d	Discuss implications of the results for practice, policy, and future research.	Discussion Relevance to Patient Care and Clinical Practice
**OTHER INFORMATION**			
Registration and protocol	24a	Provide registration information for the review, including register name and registration number, or state that the review was not registered.	Methods
	24b	Indicate where the review protocol can be accessed, or state that a protocol was not prepared.	Methods
	24c	Describe and explain any amendments to information provided at registration or in the protocol.	NA
Support	25	Describe sources of financial or nonfinancial support for the review, and the role of the funders or sponsors in the review.	Funding
Competing interests	26	Declare any competing interests of review authors.	Declaration of interests
Availability of data, code, and other materials	27	Report which of the following are publicly available and where they can be found: template data collection forms; data extracted from included studies; data used for all analyses; analytic code; any other materials used in the review.	NA

Abbreviation: NA, not applicable.

**Table A2.** Ovid MEDLINE Search Strategy: Epub Ahead of Print, In-Process and Other Nonindexed Citations, Ovid MEDLINE Daily and Ovid MEDLINE 1946-Present (Original Search February 2020 Before Update in November 2021).

#	Searches	Results
1	Critical Care/	51 016
2	intensive care units/ or burn units/ or coronary care units/ or intensive care units, pediatric/ or recovery room/ or respiratory care units/	67 942
3	(ICU or ICUs or PICU or PICUs or SICU or SICUs or CCU or CCUs).tw, kf,kw.	62 411
4	([ or critical or acute] adj3 care).tw, kf,kw.	190 858
5	([ or coronary or heart] adj3 (unit$1 or center$1 or center$1)).tw, kf,kw.	12 640
6	(respiratory adj3 [unit$1 or center$1 or center$1]).tw, kf,kw.	3692
7	([ or surger$] adj3 (unit$1 or center$1 or center$1)).tw, kf,kw.	21 708
8	(burn adj3 [unit$1 or center$1 or center$1]).tw, kf,kw.	4184
9	Ketamine/	12 186
10	(ketamine$ or calipsol$ or calypsol$ or kalipsol$ or ketalar$ or ketanest$ or ketaset$).tw, kf,kw.	18 220
11	“Hypnotics and Sedatives”/	28 858
12	Deep Sedation/ or Conscious Sedation/	9700
13	Anesthesia, Intravenous/ or Anesthesia/ or “Anesthesia and Analgesia”/	72 342
14	Anesthetics/ or Anesthetics, General/	23 073
15	Sedat$.tw, kf,kw.	59 329
16	($sedation or $sedations or $sedate or $sedates or $sedatory or $sedative or $sedatives).tw, kf,kw.	55 125
17	(anesthe$ or anaesthe$).tw, kf,kw.	380 063
18	1 or 2 or 3 or 4 or 5 or 6 or 7 or 8	273 211
19	9 or 10	19 673
20	11 or 12 or 13 or 14 or 15 or 16 or 17	453 017
21	18 and 19 and 20	418
22	animals/ not humans/	4 640 038
23	21 not 22	387

Abbreviations: CCU, critical care unit; ICU, intensive care unit; PICU, pediatric intensive care unit; SICU, surgical intensive care unit.
